# Identifying opportunities for shared decision-making through patients’ and physicians’ perceptions on the diagnostic process: A qualitative analysis of malpractice claims in general practice

**DOI:** 10.1080/13814788.2025.2501302

**Published:** 2025-06-02

**Authors:** Sofie Jacobse, Hanneke Rijkels-Otters, Manon Eikens-Jansen, Trudy van der Weijden, Glyn Elwyn, Walter van den Broek, Patrick Bindels, Laura Zwaan

**Affiliations:** ^a^ Department of General Practice, Erasmus Medical Center Rotterdam, Rotterdam, the Netherlands; ^b^ Institute of Medical Education Research Rotterdam (iMERR), Erasmus Medical Center Rotterdam, Rotterdam, the Netherlands; ^c^ VvAA Group, Utrecht, the Netherlands; ^d^ Department of Family Medicine, Care and Public Health Research Institute CAPHRI, Maastricht University, Maastricht, the Netherlands; ^e^ The Dartmouth Institute for Health Policy and Clinical Practice, Lebanon, PA, USA

**Keywords:** General practice, shared decision making, diagnostic testing, malpractice claims

## Abstract

**Background:**

Shared decision-making (SDM) is considered the preferred communication model, yet its applicability in the diagnostic process is understudied.

**Objective:**

To identify clinical situations in the diagnostic process that could benefit from SDM.

**Methods:**

An observational study of closed malpractice claims against general practitioners (2012–2020) related to problems of diagnosis, obtained from a liability insurance company in the Netherlands. We established SDM-selection criteria, specified for the diagnostic process (i.e. diagnostic uncertainty, multiple options and clinical equipoise). Phase 1: We selected and categorised eligible cases, using summarised information from a claim database. Phase 2: We analysed 90 fully documented claims and extracted information from GPs and patients related to the diagnostic process. Using this data, we conducted an inductive thematic analysis.

**Results:**

Phase 1: 261 out of 1477 claims (18%) met the SDM-selection criteria. The main reason for complaints was (omitted) test-ordering (155 claims, 59.4%). The most frequent final diagnoses were: fracture (49%), malignancy (10%), infection (9%), tendon rupture (8%) and cardiovascular disease (4%). Phase 2: Six types of diagnostic considerations emerged from the data: diagnostic uncertainty, using time as a diagnostic tool, management consequences, information about test indication or procedure, indications for re-evaluation and individual patient context. Contradictory statements from GPs and patients demonstrated a lack of shared understanding.

**Conclusion:**

The diagnostic process could benefit from SDM in several areas, including discussing diagnostic options, test conditions (e.g. timing and procedure) and follow-up. SDM training programs should be tailored to encourage clinicians to apply SDM in diagnostic decisions.

## KEY MESSAGES

Malpractice claim data from the Netherlands suggest a significant role for shared decision-making in the diagnostic process.

Contradictory perspectives from patients and GPs, e.g. confusion over patient consent for an investigation, were indicative of a lack of shared understanding.

The use of SDM for discussing diagnostic options, circumstances of testing and follow-up should be encouraged.

## Introduction

Shared decision-making (SDM) aims to enhance medical decision-making through increased patient engagement and improved communication. In this model, the physician and the patient jointly make decisions, combining evidence with personal preferences [1]. The merits of SDM have been established in treatment and screening, but are less studied in diagnostics. This is evident from a Cochrane review on patient decision aids, which featured only five studies on diagnostic decisions, compared to 261 and 71 studies on treatment and screening, respectively [2]. SDM is fundamentally different in diagnostic decisions: actions or deliberate inactions following the assessment of a patient’s health complaint or concern, for which there is not yet a diagnosis (such as active surveillance, testing or referral). Greater uncertainty in these decisions makes it more challenging to involve patients and to predict if someone will benefit from investigations [3]. Tests may identify treatable conditions, but also expose patients to downstream consequences, such as overdiagnosis and overtreatment [4,5]. Physicians may fear that displaying uncertainty will be misinterpreted as incompetence [6]. Patients may overestimate test accuracy and fail to recognise limitations [7,8]. These challenges contribute to the relative neglect of SDM in the diagnostic process.

Yet, considering the impact of diagnostic errors and the costs and risks of overtesting, the relevance of SDM in the diagnostic process is irrefutable [9]. Diagnostic errors are the most common and costly malpractice claims with severe consequences for patients [10]. Communicating uncertainty can improve patient satisfaction and reduce the risk of errors [11]. To advance the quality and safety of care, active patient involvement in diagnostic decisions is increasingly advocated by health professionals, patients and other stakeholders [3,9]. It is considered particularly relevant in general practice, where diagnostic uncertainty is high and the risk of serious disease is low [12,13]. Not all situations are equally suitable for SDM. It is considered most relevant in preference-sensitive decisions that contain multiple options, a high level of uncertainty and clinical equipoise [14,15]. Identifying these type of decisions may help the adoption of SDM in the diagnostic process. In this study we reviewed diagnostic malpractice claims in general practice that contained such preference-sensitive decisions. Claim data provide insight into potential high impact situations that have caused patient dissatisfaction. They contain perspectives from patients and health care providers. A mismatch of expectations is one of the main reasons for filing a complaint [16,17]. The objective of this study was to identify clinical situations in the diagnostic process where SDM may be of value. We aimed to answer the following research questions:What are characteristics of diagnostic malpractice claims with suitable conditions for SDM?Which topics are addressed by GPs and patients within these claims?Which underlying diagnostic considerations can be identified within these claims?

## Methods

This retrospective descriptive study consists of two phases. In phase 1 we used a pseudonymised malpractice claim database of the largest liability insurance company for general practitioners (GPs) in the Netherlands to select and describe potentially relevant cases for SDM. This database contains summarised information of all closed malpractice claims that were filed against GPs between January 2012 and December 2020. In phase 2 we used purposive sampling to select 90 claims from the database for qualitative review of the corresponding anonymised claim records.

### Phase 1: Claim database

The claim database was used to select and describe relevant cases for SDM (research question 1). This database is primarily used for internal data management of the liability insurer and is filled by medical coders, based on the original claim records. For every claim it contains a brief case summary and a large set of variables, including legal characteristics, patient demographics, GP characteristics and medical details. We obtained access to a subset of this database, containing all claims that were classified by medical coders as related to the diagnostic process.

#### Case selection

We established SDM-criteria based on literature [15], specified to the diagnostic process, to select claims that contained a preference-sensitive decision:

- There is considerable uncertainty regarding the medical diagnosis.
- There is a choice out of two or more diagnostic decisions, which may include active surveillance.
- There is no consensus in literature on the preferred diagnostic option, or the situation allows for deviating from the existing guidelines.

Claims were eligible regardless of whether liability was acknowledged or not. For cases with complaints against more than one GP we included the claim that had been received first by the insurance company. Cases concerning a psychiatric diagnosis were excluded, as it was hypothesised that these claims reflect more complex communication challenges. Two authors with a medical background, one GP (HR) and one GP trainee (SJ), independently applied the selection criteria to all case summaries. The first 50 claims were discussed together to ensure alignment between the raters. The remaining claims were assessed independently. Discrepancies were discussed until consensus was reached and inter-rater reliability (IRR) was calculated using an intra-class correlation coefficient (ICC).

#### Data processing and analysis

The following variables were extracted from the database: patient age and gender, diagnostic step against which the complaint was direct (e.g. clinical examination), final diagnosis and the judicial judgement. Final diagnoses were categorised into chapters and codes of the International Classification of Primary Care (ICPC-3) [18]. Excel software (2016) was used to obtain descriptive statistics.

### Phase 2: Claim records

An in-depth analysis was performed with data from 90 anonymised claim records to identify themes relevant for SDM. They contained all types of correspondence between the insurer and the patient, GP or legal representative, including medical documents. Based on existing guidelines for risk analysis [19,20], we had estimated that a minimum of 50 claim records would be needed for the analysis. Upon data collection it was perceived that more claims were needed to ensure that no new (sub)categories were identified. After analysing 40 additional claim records, data saturation was reached.

#### Case selection

Using a purposive sampling method, we selected claims from all ICPC-3 codes in the database to ensure a diverse sample. When multiple cases were available for the same code, we selected claims with the most detailed description of the GPs and patients perspective in the claim summary.

#### Data processing and analysis

One author (SJ) reviewed the claim records and extracted all documents with content-specific remarks from patients, GPs and their legal representatives. The data were analysed in NViVo 14 using inductive thematic content analysis [21] to identify emerging themes and patterns.

### Step 1: General overview of topics (research question 2)

In the first step of data analysis, the aim was to create an overview of topics that were addressed by GPs and patient in the claims. One author (SJ) generated initial descriptive codes using the full dataset. Codes were attached to semantic meanings of the data [22], in order to stay close to the explicit issues addressed by patients and GPs. Next, codes were grouped with axial coding by one author (SJ) to find relationships between categories and subcategories. The coding scheme was constantly reviewed and refined in close collaboration with two other researchers (HR and LZ) during weekly data sessions. Links between categories were highlighted and themes and subthemes were identified. The themes were discussed and refined within the core research team until consensus was reached.

### Step 2: Identifying themes of diagnostic considerations (research question 3)

Upon data analysis it was observed that a subset of 59 cases contained substantive arguments in favour or against a diagnostic decision. It was decided to recode these items to latent meanings of the data [22], to gain a deeper understanding of the type of arguments used in diagnostic decisions. The same procedure was followed as described under step 1. Themes were further explored during regular meetings within the research team and used to formulate underlying decision types. The decision types were applied to the entire dataset and further developed into a model using recurring case characteristics.

## Results

### Phase 1: Claim database

#### Case selection

1477 diagnostic claims were reviewed using the previously described selection criteria. Out of the eligible claims, 267 cases were selected as potentially relevant topics for SDM. Six claims concerning a psychiatric diagnosis were excluded. Therefore, 261 claims (18%) were included in the analysis. The inter-rater reliability on the selection of cases was substantial (94%, k 0.77).

#### General characteristics

General patient, GP and consultation characteristics are presented in Table 1. The majority of complaints concerned test ordering (155 claims, 60%), as compared to history taking and clinical examination (55 claims, 21%), interpretation of findings (7 claims, 3%) and next diagnostic steps (41 claims, 16%). Within this category, most patients complained about omitted tests (137 claims, 88%), as compared to unnecessary or incorrect tests (18 claims, 12%). This finding indicates that test ordering, and particularly the decision to refrain from testing, is an important topic to discuss with patients during consultations. In 233 cases (89%) the complaint involved a patient reported missed or delayed diagnosis. More than half of these perceived errors were fractures (128 claims, 55%), followed by malignancies (27 claims, 12%), infections (23 claims, 10%), tendon ruptures (20 claims, 9%) and cardiovascular diseases (11 claims, 5%). The remaining 28 claims without a diagnostic error were filed due to financial harm (27 claims) or emotional harm (1 claim), resulting from diagnostic tests or referrals deemed unnecessary from the patient’s perspective. Categorisation of final diagnoses into the International Classification of Primary Care 3 (ICPC-3) showed that 90 percent of cases concerned the musculoskeletal, digestive, genital, neurological, circulatory and respiratory system. Final diagnoses were categorised into 53 codes of the ICPC-3 (Supplementary Appendix 1). These findings highlight health topics for which SDM may be relevant.

**Table 1.** General claim characteristics: patient demographics and complaint characteristics.

**Patient’s age**	All claims *n* = 261
Mean age (SD)	41.5 (19.1)
Unknown age (%)	29 (11.1)
**Patient’s sex**	*n* (%)
Male	116 (44.4)
Female	126 (48.3)
Unknown	19 (7.3)
**Diagnostic step** ^a^	*n* (%)
History taking and clinical examination	55 (21.1)
Diagnostic test ordering	155 (59.4)
Interpretation of findings	7 (2.7)
Next diagnostic steps	41 (15.7)
Unknown	3 (1.2)
**Patient reported diagnostic errors** ^b^	*n* (%)
Fracture	128 (54.9)
Malignancy	27 (11.6)
Infection	23 (9.9)
Tendon rupture	20 (8.6)
Cardiovascular disease	11 (4.7)
Other errors	24 (10.3)
Total errors	233 (100)
**Other complaint causes** ^c^	*n* (%)
Financial harm	27 (96.4)
Emotional harm	1 (3.6)
Total	28 (100)
**Categorisation of final diagnoses** ^d^	*n* (%)
Musculoskeletal system	161 (61.7)
Digestive system	24 (9.2)
Genital system	17 (6.5)
Neurological system	12 (4.6)
Circulatory system	8 (3.1)
Respiratory system	5 (1.9)
Skin	3 (1.1)
Endocrine system	1 (0.4)
Urinary system	1 (0.4)
General and unspecified	1 (0.4)
Unknown	28 (10.7)

^^a^^ Step of the diagnostic process that the patient was dissatisfied about as reported in the claim database.

^b^ The final diagnosis that was initially missed as reported in the claim database.

^c^ The main reason for the complaint in cases without a reported diagnostic error.

^d^ Final diagnoses were categorised by the research team into chapters of the ICPC-3.

### Phase 2: Claim records

#### Topics addressed by patients and GPs

Table 2 presents the identified main themes and subthemes. Nearly half of the codes (49%) concerned diagnostic management, highlighting steps in the diagnostic process that were perceived as (in)sufficient. The remaining codes (51%) addressed non-medical aspects relevant to SDM: professionalism, communication and decision-making. A lack of shared understanding was frequently seen throughout the dataset. For example, in case 1305 the patient complains that the GP did not perform a neurologic examination (‘I asked for a neurological examination two or three times’), while the GP replies that it had been performed (‘The neurological examination showed no abnormalities’). More examples are presented in Table 2.

**Table 2.** Overview of general main themes and subthemes.

	Subtheme	Exemplary quotations (case identifier)
Main theme: diagnostic management (*n* = 168, 49%)		
Patient	Diagnostic error	‘He made a wrong diagnosis’. (591)
	Insufficient clinical examination	‘I asked for a neurological examination 2 or 3 times’. (1305)
	Insufficient test ordering	‘The GP unjustly did not order an X-ray’. (1239)
	Insufficient referral	‘The GP refused to refer me to a specialist’. (772)
	Incorrect medical advice	‘I have been wrongly advised’. (213)
	Incorrect treatment	‘The GP gave me the wrong treatment’. (192)
GP	No errors in medical management	‘I do not think that I’ve made a mistake in diagnosis or treatment’. (591)
	Adequate clinical examination	‘The neurological examination showed no abnormalities’. (1305)
	Adequate test-ordering	‘There was no medical indication for an X-ray’. (1239)
	Adequate referral	‘I advised against a referral as I didn’t think it would be useful’. (772)
	Correct medical advice	‘I informed the patient and gave instructions on when to come back’. (630)
	Correct treatment	‘The treatment was also effective in case of a fracture’. (213)
	Following guidelines	‘I followed the Ottawa Ankle rules. An X-ray was not indicated’. (1239)
Main theme: professionalism (*n* = 99, 28%)		
Patient	Not being listened to	‘The doctor did not want to listen to my whole story’. (1089)
	Not taken seriously	‘My complaints have not been taken seriously’. (292)
	Acting negligently	‘Many of my complaints were not noted in the medical file’. (292)
GP	Listening carefully	‘Every time I listened to the patient and ordered tests at his request’. (1089)
	Taking patient seriously	‘Every time I took her complaints seriously’. (292)
	Being careful and thorough	‘I have performed a thorough clinical examination’. (419)
	Acting in the patient’s interest	‘I didn’t want to burden the patient because she was so afraid’. (976)
	Owning up to mistake	‘I immediately admitted my mistake and I did everything I could to rectify it’. (1089)
Main theme: communication (*n* = 45, 13%)		
Patient	Concerns expressed by patient	‘I came back three times because I didn’t trust it’. (116)
	Not called back	‘The doctor never called us to talk about it’. (157)
GP	No signs of patient dissatisfaction	‘The patient never indicated that she disagreed or that she had any doubts’. (304)
	Could not reach patient	‘I’ve tried to call the patient several times but I had a wrong number’. (441)
	Miscommunication	‘It’s possible that the language barrier caused miscommunication’. (52)
	Feeling pressured by patient	‘The family has always been verbally aggressive, which may have made me hesitant to reach out’. (1089)
Main theme: decision-making (*n* = 33, 10%)		
Patient	No consent given	‘I told her that I didn’t agree with the examination’. (304)
	Dismissing patient’s preferences	‘I’ve requested an X-ray several times, but he refused to order one’. (580)
GP	Discussing options with patient	‘I’ve discussed the options with the patient on two occasions’. (157)
	Shared decision-making	‘Together we decided to wait for further recovery’. (157)
	Following patient’s preference	‘It was the patient’s preference to wait-and-see’. (717)

#### Diagnostic decision types

Six types of arguments about diagnostic decision-making were identified (Table 3).

**Table 3.** Themes and subthemes of diagnostic considerations.

	Subtheme	Exemplary quotations (case identifier)
Main theme 1: diagnostic (un)certainty		
Patient	Failure to communicate level of uncertainty	‘I find the doctors’ certainty confronting. That is simply not possible (and it turned out to be wrong in my case)’. (888)
GP	Low probability of serious diagnosis	‘Appendicitis was not high on my list of differential diagnoses, based on the patients’ symptoms’. (527)
	Absence of alarm symptoms	‘There were no alarm signs and there was a reasonable explanation for the patients’ complaints’. (1091)
Main theme 2: the use of time as a diagnostic tool		
Patient	No advice given on when to come back	‘The GP never explained that I had to come back if my symptoms persisted. As a result I went on with that fracture for a very long time’. (985)
GP	Instructing patient when to come back (safety-net advice)	‘According to the guidelines there was no need for an X-ray at that time. I explained my findings and I gave safety-netting advice’. (779)
	Awaiting disease course over time helps in diagnostic assessment.	‘The resident wanted to await the disease course over time. Upon reassessment by telephone the situation had not deteriorated’. (411)
Main theme 3: management consequences of diagnostic work-up		
Patient	Preferring a test for reassurance of a benign cause	‘My mother-in-law, who is also a physician, recognised the disease and recommended a blood test, but the GP refused to listen. Because of this, I have had unnecessary fear of cancer’. (1089)
Patient	Preferring a test to prevent costs for unnecessary treatment	‘The GP made a wrong diagnosis. As a result, I had to undergo incorrect treatment with multiple sessions of physiotherapy’. (591)
GP	Refrain from testing when there are no consequences for therapeutic management	‘My conclusion was that the toe was broken. The patient wanted an X-ray, but it would not change the management. Therefore, I didn’t order one’. (752)
Main theme 4: need of sufficient information about test indication or procedure		
Patient	Insufficient information given about the indication for a test.	‘I was informed incorrectly about the reason for the cervical smear. Because of this, I could not decide for myself whether I wanted the test. I am shocked that I was not informed in advance about the abnormal findings’. (349)
	Insufficient information given about the procedure and costs of a test.	‘If I had known that the test had to be performed in the hospital, I would have preferred to wait a little longer. I expected all GP care to be free of charge’. (272)
Main theme 5: indication for re-evaluation of a working diagnosis		
Patient	Persisting or progressing symptoms	‘When my symptoms persisted, the GP should have run more tests, even though the X-ray was normal’. (206)
GP	Reassuring test results and/or good treatment response	‘The X-ray was normal and the patient responded well to physiotherapy. There was no reason to reconsider the diagnosis’. (206)
Main theme 6: individual patient context		
Patient	Cultural background not taken into account	‘I have repeatedly told her that I could not accept the examination. The GP failed to take my cultural background into account’. (304)
	Medical or family history justifies deviating from guidelines	‘The GP should have taken into account that he is dependent on his right leg because he has a prosthesis on his left leg’. (580)
	Patients’ occupation justifies deviating from guidelines	‘A good wrist function is essential in my profession. The GP failed to take this into account sufficiently’. (580)

### Theme 1: Diagnostic (un)certainty

This theme arose in cases with likely benign health complaints that required invasive or costly tests to rule out serious conditions, such as patients with chronic headaches and a normal neurological exam. GPs justified not pursuing further testing when there were no alarm symptoms and the risk of serious disease was low. Patients complained that they had been falsely reassured by the doctors certainty. They claimed that the uncertainty of a diagnosis should have been communicated.

### Theme 2: The use of time as a diagnostic tool

This theme was observed when GPs expected patients’ symptoms to improve quickly, such as in cases of acute abdominal pain and a normal pelvic exam. GPs justified watchful waiting in the absence of alarm signs, and only recommended further tests if symptoms persisted or progressed. They considered time a valuable diagnostic tool, as long as follow-up was arranged and patients were informed about alarm signs. Patients complained about insufficient follow-up. They were not always told the indications for a return visit, which had contributed to a delayed or missed diagnosis.

### Theme 3: Management consequences of diagnostic work-up

This theme was seen in cases where a definitive diagnosis would not change the management of a patients symptoms. For example, patients with acute knee injury without the suspicion of fractures or other serious injury. GPs explained that testing was unnecessary, because the outcome (e.g. contusion, small meniscal tear) would not change treatment. Patients often asked for additional tests. They considered reassurance and exclusion of a serious diagnosis also important indications for a diagnostic test.

### Theme 4: Insufficient information about test indication or procedure

This theme was observed when patients were not informed sufficiently about the indication or procedure of a test, such as a cervical pap smear. Patients complained that a lack of information prevented them from making a deliberate decision. Additionally, many patients were displeased about the costs of a test and claimed that the GP should have informed them beforehand. This theme was not observed in the GP data.

### Theme 5: Indications for re-evaluation of the working diagnosis

This theme arose in cases where the initial test results were negative. For instance, a patient with chronic low back pain and a normal X-ray received was initially reassured, but later diagnosed with cancer metastases. Patients complained that the GP held on too long to the initial diagnosis. They mentioned persisting symptoms or concerns as reasons for reassessment. GPs explained that there had been no reason to re-evaluate the diagnosis, because test results were reassuring or because there was a good response to treatment.

### Theme 6: Individual patient context

Many patients felt it was necessary to deviate from guidelines due to their personal circumstances. For example, a patient expressed discomfort with a gynaecological examination because of her cultural background. Other factors that were commonly mentioned were medical or family history and occupation. They felt that their personal situation should have been taken into account, instead of merely adhering to protocols. This theme was not observed in the GP data.

#### Integration of decision types in the diagnostic process

**Figure 1.**
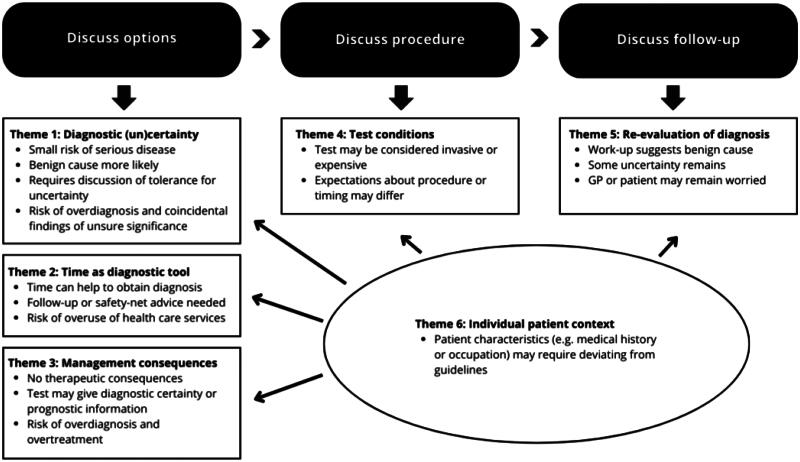
Characteristics and timing of diagnostic decision types for SDM.

The identified themes correspond to decisions made at three stages in the diagnostic process discussing diagnostic options, test conditions and follow-up. Themes 1, 2 and 3 involve choosing between different diagnostic options, which requires weighing benefits against risks. While GPs did not explicitly mention risks, they are widely recognised: invasive tests for a small disease risk may cause overdiagnosis and overtreatment (Theme 1), acting too soon when symptoms may resolve lead to overuse of health care services (Theme 2), and testing without clinical consequences may lead to both issues. Theme 4 reflects decisions about test conditions, such as when and how to perform a test. This decision comes after a choice has been made out of multiple diagnostic options. Factors like cost, location, or procedure may be of influence. For instance, a GP might consider a gynaecological exam standard procedure for female patients with acute abdominal pain, while the patient may prefer to postpone it to a follow-up visit. Theme 5 comprises the discussion if and when the diagnosis should be re-evaluated. Often there remains some uncertainty after a diagnostic work-up. Conflicting views on acceptable uncertainty require careful deliberation with the patient. Patient context (Theme 6) was observed across all decision types and phases of the diagnostic process. Characteristics and timing of the conversation types are shown in Figure 1. Table 4 provides clinical examples of the decision types based on cases from our dataset.

**Table 4.** Clinical examples^a^ of diagnostic decision types.

Theme 1: Diagnostic (un)certainty	
***Clinical example*** A 68-year-old man presenting with recurrent episodes of vertigo. The neurological examination showed no abnormalities. Symptoms are not indicative of cardiovascular disease and the EKG is normal. A benign vestibular cause is the most likely diagnosis, but serious neurologic pathology cannot be ruled out completely without invasive tests.	***What SDM can add*** Dizziness can be very troublesome, but has a benign cause in the majority of cases. In 1 to 2 out of 100 patients with dizziness there are serious abnormalities found on a brain scan [30,31]; most of these patients also have other symptoms. Explain the options of watchful waiting, supportive therapy or diagnostic referral and ask about the patients’ preferences. Weigh the benefits of finding a rare diagnosis against the harms of higher costs, invasive diagnostic tests and risk of false positive and false negative results, overdiagnosis and incidental findings of unsure significance.
Theme 2: time as diagnostic tool	
***Clinical example*** A 65-year-old woman presents with inversion trauma of the ankle. She has no known history of osteoporosis, no prior fractures or falls and her BMI is normal. Clinical examination reveals some swelling and a malleolar haematoma. The Ottawa Ankle Rules (OAR) are negative. The GP hesitates whether to order an X-ray.	***What SDM can add*** Based on the examination an X-ray is not strictly indicated, as less than 1 in 1000 patients with negative OAR have an ankle fracture [32]. However, her age, gender and the haematoma increase the prior fracture risk. Discuss the options of watchful waiting or a referral to the hospital for an X-ray. Assess the patients’ preferences regarding diagnostic certainty. Weigh the costs and inconvenience of testing against the risk of a missed diagnosis.
Theme 3: Management consequences	
***Clinical example*** A 42-year-old woman presents with lower back pain without radiation. She has no history of back pain or malignancy. The neurologic examination is normal. The GP considers non-specific low back pain and proposes supportive treatment with painkillers and physiotherapy. The patient seeks diagnostic certainty and asks for a scan.	***What SDM can add*** Low back pain occurs frequently and can cause severe impairment. In 95 out of 100 patients there is no specific underlying cause (such as a fracture or inflammatory disease). Imaging tests may reveal degenerative abnormalities of unsure significance, as they are also frequently seen in people without low back pain [33,34]. Explain the test characteristics and inquire about the patients preferences. Weigh the benefits of finding a rare diagnosis against the costs and risk of false negative and positive test results, overdiagnosis and negative illness perceptions.
Theme 4: test conditions	
***Clinical example*** A 22-year-old woman presents with recurrent non-specific abdominal complaints. The abdominal and gynaecological examination show no abnormalities. Upon examination, the GP decides to take a swab test to rule out chlamydia. When the patient finds out she is displeased as she is currently not sexually active.	***What SDM can add*** Chlamydia is more than twice as prevalent in young adults [35]. Symptoms are often non-specific and may go unnoticed for a long time. Discuss the procedure and rationale for this test. Explore the patients’ perceptions on her infection risk. Weigh the costs and inconvenience of testing against unwanted complications such as subfertility. Decide together with the patient if and when the test should be performed.
Theme 5: re-evaluation of diagnosis	
***Clinical example*** 34-Year-old man who has previously been diagnosed with irritable bowel syndrome (IBS) presents with persistent abdominal pain and diarrhoea. There is no family history of colorectal diseases, inflammatory markers are not elevated, and coeliac screening is negative. So far, dietary measures and medication have had no effect. The patient fears cancer and requests a colonoscopy.	***What SDM can add*** In less than 1 in 1000 patients without alarm signs a tumour was found upon colonoscopy [36,37]. Also, many patients are not reassured after the test is negative [38]. Explain that IBS is the most likely diagnosis, but that you should decide together when the diagnosis needs to be re-evaluated. Explain that dietary changes are difficult and may need time to prove their effect. Explore the reason behind the patient’s concern and discuss his attitude to risk. Weigh the advantages of finding a rare disease against the harms of invasive diagnostic tests and complication risks.

^a^ Characteristics of multiple cases were combined to ensure that the examples cannot be traced back to individuals.

## Discussion

This study aimed to identify suitable situations for SDM within the diagnostic process. We screened 1477 claims against GPs using predefined SDM-criteria. Almost one in 5 cases (18%) across various health topics was identified as potentially relevant for SDM.

Perspectives from patients and GPs on the diagnostic management, communication, professionalism and decision-making were generally conflicting, suggesting a lack of shared understanding. We identified five types of diagnostic decisions that require careful deliberation with patients.

To our knowledge, this is the first study using empirical data to study the role of SDM in the diagnostic process. Collaboration with the largest liability insurer in the Netherlands and a study period of 10 years ensured a large and diverse sample of diagnostic claims. The in-depth claim analysis provided a unique insight into the perceptions of patients and GPs on the diagnostic process. A limitation of this data source is its complex generalisability to daily practice. Malpractice claims only represent a small fraction of significant errors with extreme outcomes and provide limited insight into occurrence of overdiagnosis. To address this limitation we selected cases based on presenting symptoms, which align more closely with routine practice, and we included cases regardless of the outcome. However, due to the subjective nature of the selection criteria, some relevant cases may have been overlooked. Therefore, this study does not claim to provide a comprehensive overview of all situations where SDM can be applied. Instead, it aims to describe clinically relevant situations where SDM may be of merit. Nonetheless, the decision types unravelled in this study are reflective of the complex decisions that are being made in general practice on a daily basis. Hence, we feel that they are applicable to a broad range of diagnostic decisions in general practice and possibly in other specialties.

Our findings indicate that a wide variety of topics that are frequently seen in general practice could benefit from SDM, including health complaints from the musculoskeletal, digestive and genital system. This is in agreement with previous studies that have emphasised the relevance of SDM in the diagnostic process [3,9,23]. Several exemplary quotations from patients and GPs demonstrate a striking misalignment of their views on the decision-making process, which has also been reported in previous studies [24,25]. Although the most common diagnostic errors in this study are also frequently seen in other malpractice claim studies [13,26], the relatively high proportion of musculoskeletal disorders is remarkable. This finding may be partially explained by the high prevalence of musculoskeletal symptoms in general practice [27] and malpractice studies [28]. Moreover, other frequent diagnoses in malpractice claims, such as cardiovascular events and infections [29], often concern emergency settings that lack clinical equipoise. These topics were less likely to meet our inclusion criteria and are therefore expected to be underrepresented in this study. In this study, we identified clinical topics and decision types within the diagnostic process where SDM can be of value. We hypothesise that SDM will enhance patients’ choice awareness and comprehension of downstream consequences of a diagnostic work-up. Future research is needed to examine how SDM can be used within the diagnostic in practice. Barriers and facilitators should be explored. We advocate for the integration of diagnostic examples into the curriculum of SDM training programs and encourage physicians to look for diagnostic opportunities for SDM. The decision types may serve as an inspiration to identify these opportunities and can be used in SDM training programs.

## Conclusion

Our findings suggest that several areas within the diagnostic process can benefit from SDM, including discussing diagnostic options, test circumstances (e.g. timing and procedure) and follow-up. SDM training programs should be tailored to encourage clinicians to apply SDM in diagnostic decisions. This study marks a first but significant step towards the integration of SDM in diagnostic decisions, both within the consultation room and as part of research endeavours.

## Supplementary Material

Supplemental Material

## References

[1] Charles C, Gafni A, Whelan T. Shared decision-making in the medical encounter: what does it mean? (or it takes at least two to tango). Soc Sci Med. 1997;44(5):681–692. doi: 10.1016/s0277-9536(96)00221-3. 9032835

[2] Stacey D, Légaré F, Lewis K, et al. Decision aids for people facing health treatment or screening decisions. Cochrane Database Syst Rev. 2017;4(4):CD001431. doi: 10.1002/14651858.CD001431.pub5. 28402085 PMC6478132

[3] Berger ZD, Brito JP, Ospina NS, et al. Patient centred diagnosis: sharing diagnostic decisions with patients in clinical practice. BMJ. 2017;359:j4218. doi: 10.1136/bmj.j4218. 29092826

[4] Martin SA, Podolsky SH, Greene JA. Overdiagnosis and overtreatment over time. Diagnosis. 2015;2(2):105–109. doi: 10.1515/dx-2014-0072. 29540020

[5] Tuut MK, Burgers JS, van der Weijden T, et al. Do clinical practice guidelines consider evidence about diagnostic test consequences on patient-relevant outcomes? A critical document analysis. J Eval Clin Pract. 2022;28(2):278–287. doi: 10.1111/jep.13619. 34553815 PMC9292948

[6] Katz J. Why doctors don’t disclose uncertainty. Hastings Center Rep. 1984;14(1):35–44. doi: 10.2307/3560848. 6715153

[7] van Bokhoven MA, Pleunis-van Empel MC, Koch H, et al. Why do patients want to have their blood tested? A qualitative study of patient expectations in general practice. BMC Fam Pract. 2006;7(1):75. doi: 10.1186/1471-2296-7-75. 17166263 PMC1769380

[8] Meyer AND, Giardina TD, Khawaja L, et al. Patient and clinician experiences of uncertainty in the diagnostic process: current understanding and future directions. Patient Educ Couns. 2021;104(11):2606–2615. doi: 10.1016/j.pec.2021.07.028. 34312032

[9] Zwaan L, El-Kareh R, Meyer AND, et al. Advancing diagnostic safety research: results of a systematic research priority setting exercise. J Gen Intern Med. 2021;36(10):2943–2951. doi: 10.1007/s11606-020-06428-3. 33564945 PMC8481519

[10] Tehrani ASS, Lee H, Mathews SC, et al. 25-Year summary of US malpractice claims for diagnostic errors 1986–2010: an analysis from the National Practitioner Data Bank. BMJ Qual Saf. 2013;22(8):672–680. doi: 10.1136/bmjqs-2012-001550. 23610443

[11] Dahm MR, Crock C. Understanding and communicating uncertainty in achieving diagnostic excellence. JAMA. 2022;327(12):1127–1128. doi: 10.1001/jama.2022.2141. 35238876

[12] Alam R, Cheraghi-Sohi S, Panagioti M, et al. Managing diagnostic uncertainty in primary care: a systematic critical review. BMC Fam Pract. 2017;18(1):79. doi: 10.1186/s12875-017-0650-0. 28784088 PMC5545872

[13] Singh H, Giardina TD, Meyer AND, et al. Types and origins of diagnostic errors in primary care settings. JAMA Intern Med. 2013;173(6):418–425. doi: 10.1001/jamainternmed.2013.2777. 23440149 PMC3690001

[14] Elwyn G, Frosch D, Thomson R, et al. Shared decision making: a model for clinical practice. J Gen Intern Med. 2012;27(10):1361–1367. doi: 10.1007/s11606-012-2077-6. 22618581 PMC3445676

[15] van der Horst DEM, Garvelink MM, Bos WJW, et al. For which decisions is Shared Decision Making considered appropriate? – a systematic review. Patient Educ Couns. 2023;106:3–16. doi: 10.1016/j.pec.2022.09.015. 36220675

[16] Kravitz RL, Callahan EJ, Paterniti D, et al. Prevalence and sources of patients’ unmet expectations for care. Ann Intern Med. 1996;125(9):730–737. doi: 10.7326/0003-4819-125-9-199611010-00004. 8929006

[17] Hanganu B, Iorga M, Muraru I-D, et al. Reasons for and facilitating factors of medical malpractice complaints. What can be done to prevent them? Medicina. 2020;56(6):259. doi: 10.3390/medicina56060259. 32471166 PMC7353843

[18] van Boven K, Ten Napel HT, editor. ICPC-3 international classification of primary care: user manual and classification. 3rd ed. Boca Raton (FL): CRC Press; 2021.

[19] Chapman C. Project risk analysis and management—PRAM the generic process. Int J Project Manage. 1997;15(5):273–281. doi: 10.1016/S0263-7863(96)00079-8.

[20] Noord I V, Eikens MP, Hamersma AM, et al. Application of root cause analysis on malpractice claim files related to diagnostic failures. Qual Saf Health Care. 2010;19(6):e21–e21. 10.1136/qshc.2008.029801 20630930

[21] Kiger ME, Varpio L. Thematic analysis of qualitative data: AMEE Guide No. 131. Med Teach. 2020;42(8):846–854. doi: 10.1080/0142159X.2020.1755030. 32356468

[22] Braun V, Clarke V. Thematic analysis. In Cooper H, Camic PM, Long DL, editors. APA handbook of research methods in psychology. Vol. 2, Research designs: Quantitative, qualitative, neuropsychological, and biological. Washington (DC): American Psychological Association; 2012. p. 57–71.

[23] Polaris JJ, Katz JN. “Appropriate” diagnostic testing: supporting diagnostics with evidence-based medicine and shared decision making. BMC Res Notes. 2014;7(1):922. doi: 10.1186/1756-0500-7-922. 25515327 PMC4301652

[24] Amelung D, Whitaker KL, Lennard D, et al. Influence of doctor-patient conversations on behaviours of patients presenting to primary care with new or persistent symptoms: a video observation study. BMJ Qual Saf. 2020;29(3):198–208. doi: 10.1136/bmjqs-2019-009485. PMC7057803 31326946

[25] van Mook WNKA, Gorter SL, Kieboom W, et al. Poor professionalism identified through investigation of unsolicited healthcare complaints. Postgrad Med J. 2012;88(1042):443–450. doi: 10.1136/postgradmedj-2011-130083. 22595102

[26] Wallace E, Lowry J, Smith SM, et al. The epidemiology of malpractice claims in primary care: a systematic review. BMJ Open. 2013;3(7):e002929. doi: 10.1136/bmjopen-2013-002929. PMC3693415 23869100

[27] Keavy R., Horton R, Al-Dadah O. The prevalence of musculoskeletal presentations in general practice: an epidemiological study. Fam Pract. 2023;40(1):68–74. 10.1093/fampra/cmac055 35747902

[28] van Sassen CGM, van den Berg PJ, Mamede S, et al. Identifying and prioritizing educational content from a malpractice claims database for clinical reasoning education in the vocational training of general practitioners. Adv Health Sci Educ Theory Pract. 2023;28(3):893–910. doi: 10.1007/s10459-022-10194-8. 36529764 PMC10356624

[29] Newman-Toker DE, Wang Z, Zhu Y, et al. Rate of diagnostic errors and serious misdiagnosis-related harms for major vascular events, infections, and cancers: toward a national incidence estimate using the “Big Three.” Diagnosis. 2021;8(1):67–84. doi: 10.1515/dx-2019-0104. 32412440

[30] Fakhran S, Alhilali L, Branstetter B. Yield of CT angiography and contrast-enhanced MR imaging in patients with dizziness. Am J Neuroradiol. 2013;34(5):1077–1081. doi: 10.3174/ajnr.A3325. 23099499 PMC7964658

[31] Vandervelde C, Connor SEJ. Diagnostic yield of MRI for audiovestibular dysfunction using contemporary referral criteria: correlation with presenting symptoms and impact on clinical management. Clin Radiol. 2009;64(2):156–163. doi: 10.1016/j.crad.2008.08.002. 19103345

[32] Beckenkamp PR, Lin CC, Macaskill P, et al. Diagnostic accuracy of the Ottawa Ankle and Midfoot Rules: a systematic review with meta-analysis. Br J Sports Med. 2017;51(6):504–510. doi: 10.1136/bjsports-2016-096858. 27884861

[33] Jarvik JG, Deyo RA. Diagnostic evaluation of low back pain with emphasis on imaging. Ann Intern Med. 2002;137(7):586–597. doi: 10.7326/0003-4819-137-7-200210010-00010. 12353946

[34] Jensen MC, Brant-Zawadzki MN, Obuchowski N, et al. Magnetic resonance imaging of the lumbar spine in people without back pain. N Engl J Med. 1994;331(2):69–73. doi: 10.1056/NEJM199407143310201. 8208267

[35] Ribeiro AA, Saddi VA, Carneiro MA, et al. Human papillomavirus and Chlamydia trachomatis infections in adolescents and young women: prevalence and risk factors. Diagn Cytopathol. 2020;48(8):736–744. doi: 10.1002/dc.24460. 32379403

[36] Hamm LR, Sorrells SC, Harding JP, et al. Additional investigations fail to alter the diagnosis of irritable bowel syndrome in subjects fulfilling the Rome criteria. Am J Gastroenterol. 1999;94(5):1279–1282. doi: 10.1111/j.1572-0241.1999.01077.x. 10235207

[37] Tolliver BA, Herrera JL, DiPalma JA. Evaluation of patients who meet clinical criteria for irritable bowel syndrome. Am J Gastroenterol. 1994;89(2):176–178. 8304298

[38] Spiegel BMR, Gralnek IM, Bolus R, et al. Is a negative colonoscopy associated with reassurance or improved health-related quality of life in irritable bowel syndrome? Gastrointest Endosc. 2005;62(6):892–899. doi: 10.1016/j.gie.2005.08.016. 16301033

