# Supplementary material for: Identifying opportunities for shared decision-making through patients’ and physicians’ perceptions on the diagnostic process: A qualitative analysis of malpractice claims in general practice
Source: Eur J Gen Pract. 2025 Jun 2;31(1):2501302. doi: 10.1080/13814788.2025.2501302 (PMC12131537; doi:10.1080/13814788.2025.2501302)
Supplement: Supplemental Material [file IGEN_A_2501302_SM8998.docx]

**Appendix 1.** Categorization of claims into chapters and codes of the ICPC-3**.** 

| **ICPC-3 chapter** | **ICPC-3 code** | **n (%)** |
| --- | --- | --- |
| **A. General** |  |  |
|  | Other specified infectious disease (AD24) | 1 (0.4) |
|  | **Total** | **1 (0.4)** |
| **D. Digestive system** |  |  |
|  | Appendicitis (DD72) | 15 (5.7) |
|  | Malignant neoplasms of large intestine (DD26) | 5 (1.9) |
|  | Malignant neoplasms of pancreas (DD27) | 1 (0.4) |
|  | Constipation (DS12) | 1 (0.4) |
|  | Gastroenteritis presumed infection (DD05) | 1 (0.4) |
|  | Other specified or unknown abdominal hernia (DD76) | 1 (0.4) |
|  | **Total** | **24 (9.2)** |
| **G. Genital system** |  |  |
|  | Malignant neoplasms of breast (GD27) | 8 (3.1) |
|  | Malignant neoplasms of prostate (GD26) | 5 (1.9) |
|  | Malignant neoplasms of testis (GD28) | 1 (0.4) |
|  | Pelvic inflammatory disease (GD09) | 1 (0.4) |
|  | Fibromyoma of uterus  (GD29) | 1 (0.4) |
|  | Endometriosis (GD69) | 1 (0.4) |
|  | **Total** | **17 (6.5)** |
| **K Circulatory system** |  |  |
|  | Acute coronary syndrome (KD65) | 3 (1.1) |
|  | Pulmonary embolism (KD77) | 2 (0.8) |
|  | Deep vein thrombosis (KD78) | 1 (0.4) |
|  | Infection of circulatory system (KD01) | 1 (0.4) |
|  | Atherosclerosis or peripheral vascular disease (KD76) | 1 (0.4) |
|  | **Total** | **8 (3.1)** |
| **L Musculoskeletal system** |  |  |
|  | Fracture of hand or foot  (LD37) | 42 (16.1) |
|  | Fracture of tibia or fibula (LD36) | 26 (10.0) |
|  | Other specified and unknown fracture (LD39) | 27 (10.3) |
|  | Fracture of radius or ulna (LD35) | 20 (7.7) |
|  | Other specified musculoskeletal injury (LD49) | 20 (7.7) |
|  | Fracture femur (LD38) | 13 (5.0) |
|  | Back syndrome with radiating pain (LD67) | 4 (1.5) |
|  | Dislocation or subluxation (LD48) | 3 (1.1) |
|  | Injury to multiple structures of knee (LD45) | 2 (0.8) |
|  | Spondylosis (LD66) | 1 (0.4) |
|  | Congenital anomaly (AD55) | 1 (0.4) |
|  | Osteoporosis (LD81) | 1 (0.4) |
|  | Back symptom or complaint (LS02) | 1 (0.4) |
|  | **Total** | **161 (61.7)** |
| **N Neurological system** |  |  |
|  | Malignant neoplasm of nervous system (ND25) | 5 (1.9) |
|  | Stroke or cerebrovascular incident (ND69) | 4 (1.5) |
|  | Other specified and unknown disease of neurological system (ND99) | 2 (0.8) |
|  | Meningitis or encephalitis (ND02) | 1 (0.4) |
|  | **Total** | **12 (4.6)** |
| **R Respiratory system** |  |  |
|  | Malignant neoplasm of bronchus and lung (RD25) | 2 (0.8) |
|  | Pneumonia (RD09) | 2 (0.8) |
|  | Acute upper respiratory infection (RD02) | 1 (0.4) |
|  | **Total** | **5 (1.9)** |
| **S Skin** |  |  |
|  | Malignant neoplasm of skin (SD25) | 2 (0.8) |
|  | Infected finger or toe (SD05) | 1 (0.4) |
|  | **Total** | **3 (1.1)** |
| **E Endocrine system** |  |  |
|  | Other unknown endocrine disease (TD99) | 1 (0.4) |
|  | **Total** | **1 (0.4)** |
| **U Urinary system** |  |  |
|  | Urinary retention (US04) | 1 (0.4) |
|  | **Total** | **1 (0.4)** |
| **Unknown diagnosis** |  | **28 (10.7)** |
| **Total** |  | **161 (100)** |
